# Employment of the Suture in Vesico-Vaginal Fistula

**Published:** 1830-04-01

**Authors:** 


### EMPLOYMENT OF THE SUTURE FOR VESICO-VAGINAL FISTULA.*

In the last Number of this Journal we collated some valuable clinical observations on that dreadful affliction vesico-vaginal fistula, delivered in their respective schools by M. Dupuytren and Mr. Earle. The following case will not be devoid of interest to such of our surgical readers as take an interest in this class of cases. We may say that a proper acquaintance with such subjects as these is infinitely more necessary in a practical point of view, than a knowledge of what are commonly called the higher, that is the more theoretical parts of the profession. Leave to the subtle disputants of the schools, resign to the bookish theoric, disquisitions on what they call principles and laws, but let us revert to the fountain head, to the sources which give origin to theories and which crush them, to the study of *cases*, to the real investigation of disease.

*Case*. Maria Reggiani, æt. 22, had suffered since her first confinement, which had been a severe one, from vesico-vaginal fistula of such size as readily to admit the finger into the bladder. After employing various means for the space of eight months she repaired to Bologna to consult Dr. Malagodi, who performed an operation for her infirmity on the 28th of August, 1828, with the assistance of Drs. Montebagnoli and Rosaspina.

The patient was placed in the position adopted for the operation of lithotomy, and the operator introduced the forefinger of the right hand, covered with the finger-piece of a glove, into the fistulous opening. The two last phalanges of the finger were then bent like a hook, and the callous edge of the ulceration on the left side, dragged down to the orifice of the vagina. This edge was then sliced off by a semilunar incision with a straight bistoury held in the left hand. The left fore-finger was then introduced into the fistula, its callous border on the right side made to present externally, and then sliced off like the former by the bistoury held in the right hand. In order to retain the pared edges in contact, the operator provided himself with three much curved and very small needles, each armed with a thread, and mounted on a handle which might be fixed or removed at pleasure. The index finger of the right hand was then re-introduced into the opening, and its left lip again brought into view. Taking a needle mounted on its handle in the left hand, it was carried from behind forwards through the vesico-vaginal wall, near the posterior angle of the wound. The second and third needles were then passed in the same manner and at equal distances from each other,

---

* Raccoglitor Medico. Bologna. July, 1820.

and the same steps pursued on the opposite side. The ligatures were tied two and two, the lips of the wound brought into accurate contact, the patient conveyed to bed with injunctions to lie upon her back, and a catheter introduced into the bladder to prevent the urine from accumulating in the latter.

For two days the urine flowed through the instrument, but on the third, the lint retained in the vagina was found to be bathed with this fluid. On examining the condition of the parts on the following day, the two posterior ligatures were found to have answered in procuring union of portions of the wound, but the anterior ligature had cut through the parts on the left side, and consequently nearly the anterior third of the fistula was not closed. Cauterization with the nitrate of silver had been tried without success prior to the present operation, but the operator conceived that although it failed then, it might answer better now that the opening was so much diminished in calibre. Accordingly this measure was employed and the catheter constantly left in the bladder. At the end of three weeks a perceptible amelioration was perceived, and about the beginning of January the patient was declared by M. Montebagnuoli to be quite cured.

The case forms an excellent pendant to Mr. Earle's clinical lecture.

